# Analysis of risk factors for multidrug-resistant organisms in diabetic foot infection

**DOI:** 10.1186/s12902-022-00957-0

**Published:** 2022-02-21

**Authors:** Xi Yan, Jin-fang Song, Liang Zhang, Xia Li

**Affiliations:** 1 Department of Pharmacy, Changzhi People’s Hospital, Changzhi City, Shanxi Province China; 2 Department of Clinical Pharmacy, Affiliated Hospital of Jiangnan University, Wuxi City, Jiangsu Province, China; 3 Jiangsu Key Laboratory of New Drug Research and Clinical Pharmacy, Xuzhou Medical University, Xuzhou City, Jiangsu Province China; 4 Department of National Metabolic Management, Affiliated Hospital of Jiangnan University, Wuxi City, Jiangsu Province, China

**Keywords:** Diabetic foot ulcers, Diabetic food infection, Multidrug-resistant bacteria

## Abstract

**Background:**

To study the bacteriological characteristics, risk factors, and treatment of multi-drug resistance (MDR) organisms in patients with diabetic foot infection.

**Methods:**

Patients with diabetic foot ulcer admitted to hospital from June 2018 to December 2019 (*n* = 180) were selected as clinical subjects. Demographic information, routine blood test, wound culture and sensitivity were collected. Risk factors of MDR bacteria were analyzed.

**Results:**

Among 180 patients with diabetic foot ulcer, 146 were positive in bacterial culture, with 84 positive in MDR bacteria. A total of 182 strains were isolated, with 104 strains being multi-drug resistant. Body mass index, glycosylated hemoglobin, fasting blood glucose, triglyceride, course of ulcer, size of ulcer, peripheral neuropathy, peripheral vascular disease, osteomyelitis, peripheral blood leukocyte count, percentage of neutrophils, and previous use of antibiotics were the related factors of infection of MDR bacteria in diabetic foot ulcer patients (*P* < 0.05). The leukocyte count and neutrophil ratio of MDR-bacilli were lower than those of non MDR-bacilli (*P* < 0.05).

**Conclusion:**

The risk of MDR bacteria in diabetic foot infection is high. It is necessary to evaluate the risk of multidrug-resistant bacteria by characterizing the course of disease, metabolic control, local ulcer and other aspects in order to formulate an effective treatment plan. The decrease of leukocyte count and neutrophil ratio may be related to damage of the host immune response.

## Background

Diabetic patients often have a high prevalence of diabetic foot infection (DFI) due to associated neuropathy, peripheral vascular disease, immune abnormalities, etc. Some studies have shown that about 15% of diabetic patients can develop foot ulcers during their lifetime [1]. Diabetic foot ulcer wounds contain a large number of protein and carbohydrate nutrients, which can be a nidus for infection. About 40% to 80% of patients with diabetic foot suffer from a concurrent infection. 25% of patients with mild infection develops with severe deep infection. Clinically, the disease develops rapidly for a certain proportion of patients with mild infection and they need attention [2]. The types of diabetic foot infections are complex and varied. In addition to the common cellulitis, complications can involve osteomyelitis and gangrene [3]. Since the increasingly severe form of prevention and treatment of diabetic foot ulcer infections is associated with a high rate of detection of multi-drug resistant bacteria, it is important to focus on assessing the risk factors of multi-drug resistant bacterial infections in order to find more effective treatment [4–6]. Multi-drug resistance (MDR) organisms refer to bacteria that exhibit drug resistance to three or more commonly used antibiotics that are usually sensitive. Multiple drug resistance also includes extensive drug resistance (XDR) and pan-drug resistance (PDR). Clinically, Common MDR include methicillin-resistant staphylococcus aureus (MRSA), vancomycin-resistant enterococcus (VRE), bacteria that produce ultra-broad spectrum β-lactamase (ESBLs) in enterobacteriaceae (such as escherichia coli and klebsiella pneumoniae), carbapenem-resistant enterobacteriaceae, multidrug-resistant Pseudomonas aeruginosa (MDR-PA), multi-drug resistant acinetobacter baumannii (MDR-AB), etc. [7]. The aim of this study is to analyze the bacteriological characteristics of diabetic foot ulcers, drug resistance of isolates, and to reveal the potential risk factors for multi-drug resistant bacteria. This allows for more effective assessment and treatment plans for patients with MDR diabetic foot infections.

## Methods

### Study design

A total of 180 patients (96 males and 84 females) with diabetic foot ulcer admitted to our hospital from June 2018 to December 2019 were analyzed retrospectively. Their demographic information, medication history, laboratory investigations, and adverse drug reactions were collected. Inclusion criteria: (1) The diagnosis of diabetic foot was met based on relevant diagnosis and treatment guidelines [8, 9], and the diagnostic criteria of diabetic foot infection in the *Normalized and Standardized Diagnosis and Treatment of Diabetic Foot Infection* [10] were met. (2) Ulcers grading 1 to 4 based on the Wagners grading system [11]. Exclusion criteria: (1) Acute complications of diabetes mellitus other than diabetic foot ulcers. (2) Complications involving other serious cardiovascular and cerebrovascular diseases, liver and kidney insufficiency, etc. (3) Other non-diabetic foot ulcers due to vascular insufficiency, cardiac diseases, neurological disorders, malignancies, etc. This study was carried out in accordance with the recommendations of the Ethics Committee of the Affiliated Hospital of Jiangnan University (Original Area of Wuxi Third People’s Hospital) with written informed consent from all subjects.

### Collection of clinical index

Detailed history taking and physical examination was performed for each patient, and information on age,sex, height, weight, duration of diabetes, course of ulcer, site and size of ulcer, number of ulcers, use of antibiotics, and comorbidities were recorded. The size of the ulcer will be calculated by multiplying the length and width. The body mass index (BMI) was calculated by weight (kg)/height^2 (m^2). Routine blood work including fasting plasma glucose (FPG), glycated hemoglobin (HbA1c), blood pressure, total cholesterol (TC), triglyceride (TG), low-density lipoprotein (LDL-c), and high-density lipoprotein (HDL-c) were collected. X-ray, CT, or MRI examination were used to diagnose osteomyelitis.

### Microbiological detection

Specimen collection will be performed before the use of antibiotics, deep necrotic tissue or secretions from diabetic foot ulcers should be collected, and the specimens will be placed in sterilized tubes for examination within 1 h. Isolation, culture and drug sensitivity analysis of pathogenic bacteria should be performed according to standard operation procedures. VITEK-2 automatic bacterial identification system of bioMerieux (France) and its matching kit drug sensitivity card will be used for bacterial culture and drug sensitivity test.

According to China Clinical Laboratory Procedures (the Fourth Edition), isolated culture and identification of pathogenic bacteria are carried out. VITEK-2 Compact (BioMerieux bv, France) is used for bacterial identification and drug sensitive test. The minimal inhibitory concentration (MIC) dilution method is used for drug sensitive test, and the criterion of results refers to the 2016 edition relevant standards of American Institute for Clinical and Laboratory Standardization (CLSI M100-S26). Results are reported by sensitivity (S), intermediary (I), and resistance (R) [12].

### Statistical analysis

SPSS 18.0 software was used for statistical analysis. Measurement data will be expressed as mean ± standard deviation, and enumeration data will be expressed as percentage; t-test or χ^2^ test will be used for comparison of different groups, and logistic regression model will be used for multivariate analysis; *P* < 0.05 shall be considered statistically significant.

## Results

### Bacteriological condition of diabetic foot ulcer infection

A total of 180 patients with diabetic foot ulcers were included in this study, 146 (81.11%) were positive for wound culture, of which 62 (42.47%) had antibiotic-sensitive organisms and 84 (57.53%) had multidrug-resistant organisms. A total of 182 strains of bacteria were cultured, among which, 78 strains were antibiotic-sensitive (41 strains of gram-negative bacteria and 37 strains of gram-positive bacteria), 104 strains were multidrug-resistant bacteria (66 strains of gram-negative bacteria and 38 strains of gram-positive bacteria). See Table 1 for details.

**Table 1:** Bacterial Culture and Distribution of Multidrug-resistant Bacteria in Patients with Diabetic Foot ulcer infection

Bacteria	Number of sensitive strains (%)	Number of multidrug-resistant strains (%)	Total (%)
**Gram-negative bacteria**	**41(38.32)**	**66(61.68)**	**107(58.79)**
Escherichia coli	16(36.36)	28(63.64)	44(24.18)
Pseudomonas aeruginosa	15(36.59)	26(63.41)	41(22.53)
Acinetobacter	3(30.00)	7(70.00)	10(5.49)
Transmorphobacteria	3(37.50)	5(62.50)	8(4.40)
Enterobacterium	2(100)	0(0)	2(1.10)
Morganifella	2(100)	0(0)	2(1.10)
**Gram-positive bacteria**	**37(49.33)**	**38(50.67)**	**75(41.21)**
Staphylococcus aureus	19(37.25)	32(62.75)	51(28.02)
Coagulase negative staphylococcus	5(100)	0(0)	5(2.75)
Enterococcus	11(64.71)	6(35.29)	17(9.34)
β hemolytic streptococcus in group B	2(100)	0(0)	2(1.10)

### Correlation between multidrug-resistant bacteria infection and clinical indexes in patients with diabetic foot ulcer

In patients with diabetic foot ulcer infection, the correlation between infection with multi-drug resistant bacteria and various clinical indexes of patients is shown in Table 2. BMI, HbA1c, FPG, TG, ulcer course, ulcer size, peripheral neuropathy, peripheral vascular disease, peripheral blood leucocyte count, neutrophil percentage, and use of antimicrobial agents in the past 3 months were significantly different between the group of patients infected with sensitive bacteria and the group of patients infected with multiple drug-resistant bacteria(*P* < 0.05); other parameters such as sex, age, duration of diabetes, TC, HDL-c, LDL-c, Wagners' grade, hypertension, retinopathy, and nephropathy were not significantly different between the two groups of patients (P > 0.05).

**Table 2:** Comparison of clinical indexes of diabetic foot ulcer patients infected with sensitive bacteria and multidrug resistant bacteria

Index	Infected with Sensitive Bacteria(*n* = 62)	Infected with Multidrug resistant bacteria(*n* = 84)	*P* Value
Sex (M/F)	41/21	52/32	0.600
Age	50.24 ± 8.71	49.39 ± 12.29	0.643
Diabetes duration (years)	10.51 ± 6.32	9.75 ± 5.94	0.458
BMI(kg/m^2^)	24.87 ± 3.18	30.11 ± 3.61	0.000^*^
HbA1c(%)	7.91 ± 0.85	9.82 ± 0.93	0.000^*^
FPG(mmol/l)	9.35 ± 1.74	11.51 ± 1.90	0.000^*^
TG (mmol/l)	2.05 ± 1.58	2.69 ± 0.92	0.003^*^
TC (mmol/l)	4.67 ± 1.20	4.45 ± 0.88	0.203
HDL-c (mmol/l)	1.29 ± 0.45	1.31 ± 0.38	0.772
LDL-c (mmol/l)	2.79 ± 0.80	2.87 ± 0.78	0.546
Ulcer course(Month)	1.51 ± 0.92	2.20 ± 1.26	0.000^*^
Ulcer size(cm^2^)	15.21 ± 10.41	26.12 ± 14.79	0.000^*^
Wagners Grade			
I	2(3.23%)	4(4.76%)	0.644
II	25(40.32)	31(36.90%)	0.675
II	12(19.35%)	16(19.05%)	0.963
IV	23(37.10%)	33(39.29%)	0.788
Amorbidities			
Hypertension	24(38.71%)	38(45.24%)	0.430
Retinopathy	32(51.61%)	47(55.95%)	0.603
Peripheral neuropathy	25(40.32%)	57(67.86%)	0.001^*^
Kidney disease	32(51.61%)	47(55.95%)	0.603
Peripheral vascular lesions	28(45.16%)	61(72.62%)	0.001^*^
Osteomyelitis	5(8.06%)	24(28.57%)	0.002^*^
White blood cell count (*10^3^)	9.21 ± 1.87	6.51 ± 1.77	0.000^*^
Neutrophil percentage (%)	50.13 ± 17.61	38.42 ± 15.25	0.000^*^
Use of antimicrobial drugs within the last 3 months	36(58.06%)	64(78.05%)	0.020^*^

*Abbreviations*: *BMI* Body mass index, *HbA1c* Hemoglobin A1c, *FPG* Fasting plasma glucose, *TG* Triglyceride, *TC* Total cholesterol, *HDL-C* High-density lipoprotein-cholesterol, *LDL-C* Low-density lipoprotein-cholesterol

Data are expressed as mean ± SD or percentage. ^*^P < 0.05 compared with infected with sensitive bacteria group

### Analysis of multiple drug-resistant bacteria infection in patients with diabetic foot ulcer

BMI, HbA1c, FPG, TG, ulcer course, ulcer size, peripheral neuropathy, peripheral vascular lesions, osteomyelitis, peripheral blood leucocyte count, neutrophil percentage, and use of antimicrobial drugs within the last 3 months were factors associated with the presence of multi-drug resistant bacterial infections in patients with diabetic foot ulcers (*P* < 0.05), as shown in Table 3.

**Table 3:** Multi-factor Logistic Regression Analysis of Multiple Drug-resistant Bacteria Infected with Diabetic Foot Ulcer

Related Factors	β	SE	Wald	OR Value	95%CI	*P* Value
BMI(kg/m^2^)	1.572	0.883	6.342	1.309	1.214 ~ 2.419	0.021^*^
HbA1c(%)	0.734	0.619	4.529	2.013	2.104 ~ 3.872	0.008^*^
FPG(mmol/l)	1.493	0.743	7.094	1.843	1.378 ~ 2.351	0.001^*^
TG (mmol/l)	1.602	0.752	9.075	1.696	1.370 ~ 2.542	0.006^*^
Ulcer course(Month)	0.641	0.162	16.317	1.901	1.253 ~ 3.412	0.001^*^
Ulcer size(cm^2^)	0.908	0.831	10.382	2.018	1.019 ~ 3.998	0.044^*^
Perieral neuropathy	1.021	0.422	9.367	1.678	1.111 ~ 2.535	0.014^*^
Peripheral vascular lesions	1.109	0.538	12.324	1.635	1.055 ~ 2.533	0.028^*^
Osteomyelitis	1.532	0.647	8.305	1.696	1.140 ~ 2.524	0.009^*^
White blood cell count(*10^3^)	0.694	0.271	11.432	1.662	1.089 ~ 2.535	0.018^*^
Neutrophil percentage (%)	1.561	0.664	5.521	2.261	1.091 ~ 4.685	0.028^*^
Use of antimicrobial drugs within the last 3 months	0.625	0.172	14.317	1.962	1.321 ~ 3.875	0.011^*^

*Abbreviations*: *BMI* Body mass index, *HbA1c* Hemoglobin A1c, *FPG* Fasting plasma glucose, *TG* Triglyceride. ^*^P < 0.05, *P* values are determined by logistic regression model

## Discussion

This is a preliminary study of patients with diabetic foot ulcer in which each hospitalized subject was evaluated for bacteriology, clinical indexes, and antimicrobial drug use. Proper wound cleaning and debridement and collection of pus or soft tissue specimens for bacteriological culture were performed in all cases [13], and the bacteriological characteristics of diabetic foot ulcer infections and risk factors for multi-drug resistant bacterial infections were analyzed based on the culture results.

Earlier studies reported that Gram-positive bacteria such as *Staphylococcus aureus* and *Enterococcus faecalis* were considered as the most common flora of diabetic foot infections, followed by Gram-negative bacteria such as Escherichia coli and Pseudomonas aeruginosa [14, 15]. In recent years, the bacterial spectrum of diabetic foot infections has shifted considerably with the evolution of diabetes epidemiology and changes in the application of antimicrobial drugs [16, 17]. In our study, 182 strains of bacteria were cultured, including 107 strains of gram-negative aerobic bacteria, and the ratio of gram-negative bacteria to gram-positive bacteria was 1.47, which indicated that empirical antibacterial drug therapy for diabetic foot ulcer infection should pay attention to assessing the risk of gram-negative bacteria infection. *Staphylococcus aureus* was the most common bacterium among all aerobic bacteria, and 51 strains (28.02%) were cultured, which was consistent with the bacteriological characteristics of skin and soft tissue infections [18]. In the analysis of bacterial resistance, it was found that *Staphylococcus aureus* was the main multi-drug resistant bacteria in diabetic foot ulcer infection, and the resistance rate of *Escherichia coli* was the highest among gram-negative bacteria, followed by *Pseudomonas aeruginosa*, which was consistent with previous reports [19]. Infection with multidrug-resistant bacteria in diabetic foot ulcers increases the difficulty of treatment by reducing the clinical effect of antibiotics therapy, leading to amputations or deaths [5, 11]. Due to the increased resistance rate of *Escherichia coli* and *Pseudomonas aeruginosa* among gram-negative bacteria, the risk factors of drug-resistant negative bacilli in patients should be evaluated in the development of initial anti-infective treatment regimen in clinical practice. In this study, multidrug-resistant bacteria accounted for up to 57.14% of the strains obtained by wound cultures, which may be related to the patients with serious illness admitted to our hospital as a tertiary care hospital and the more complex history of antibacterial drug use.

The results of multivariate analysis indicated that BMI, HbA1c, FPG, TG, ulcer course, ulcer size, peripheral neuropathy, peripheral vascular disease, osteomyelitis, peripheral blood leucocyte count, neutrophil percentage, and use of antimicrobial agents in the past 3 months were factors associated with multidrug-resistant bacteria in diabetic foot ulcer infections. Patients infected with multidrug-resistant bacteria have higher BMI, HbA1c, FPG, and TG levels, indicating that substandard metabolic management including body weight, blood glucose, and blood lipids increases the risk of multidrug-resistant bacteria infection. Dubský and other reports show that HbA1c greater than 7.5% is an important risk factor for ulcer recurrence. The results of this study also confirmed that long-term poor blood glucose control is a factor in the emergence of multidrug-resistant bacteria [20]. The proportion of diabetic foot in patients with BMI < 25 kg/m2 was significantly increased, and low BMI was a risk factor for diabetic foot symptoms. Low BMI would lead to malnutrition of diabetic patients, and the ability to control blood sugar, blood lipid and blood pressure of elderly diabetic patients with deficient nutrition will be reduced, which will often lead to the development of diabetic foot and related complications. Diabetic patients have poor metabolic control ability. Long-term hyperglycemia leads to long-term excessive consumption of patients' bodies, atherosclerosis of blood vessels and long-term disorder of blood lipids. High TG level can affect the blood supply function of feet and aggravate infection ulcer [21].

The course of ulcer and the size of ulcer are important indicators for evaluating the wound condition of patients. The findings show that the patients with delayed ulcer healing and large ulcer wound have a greater risk of multi-drug resistant bacterial infection. The longer the wound time of diabetic foot ulcer is, the higher the Wagner grade is, and the proportion of severe infection and mixed infection of the wound increases, which may be related to the decrease of immune function during the infection of diabetic foot ulcer, which leads to the increase of drug-resistant bacteria infection. The bacterial reproduction of the wound deepens the degree of tissue damage and then prolongs the course of disease. These processes are in vicious circles [22].Patients with peripheral neuropathy and peripheral vascular disease often have sensory loss and wound disorder, which are the deep-seated causes of prolonged ulcer healing and recurrent infection. Irrational use of antibacterial drugs is closely related to bacterial resistance. This study found that the risk of multi-drug resistant bacterial infection in patients who used antibacterial drugs in the past 3 months may be greater. Multiple use of antibacterial drugs during treatment can also form a pressure screen to have an effect on bacterial resistance. Subjects with osteomyelitis often use antibacterial drugs with more types, higher intensity and longer course of treatment, which will also increase the risk of pathogen resistance to a certain extent.

The leukocyte count and neutrophil percentage were within the normal range in both groups of patients with diabetic foot ulcers infected with sensitive and multi-drug resistant bacteria, but the leukocyte count and neutrophil ratio were lower in patients infected with multi-drug resistant bacteria compared to those infected with sensitive bacteria. Both leukocytes and neutrophils have important roles in physiological and pathological processes, such as angiogenesis, hematopoiesis, wound healing, inflammation, and infectious diseases [19, 23]. The important role of neutrophils in the production and function of pro- and anti-inflammatory-related cytokines has been investigated using humans and animals as subjects, and impaired host immune responses as manifested by decreased inflammatory cell numbers and functions can affect the trauma repair process [24]. Impaired host immune response may be one of the factors associated with multi-drug resistant bacteria infection in patients with diabetic foot ulcers. Currently, no literature has been reported on the effect of multi-drug resistant bacterial infections on the immune response capacity. The treatment of diabetic foot ulcer coinfection includes systemic therapy and topical therapy, namely systemic antibiotic therapy and topical debridement. In diabetic foot ulcer coinfection, thorough debridement is the basis for successful treatment of diabetic foot ulcer. When patients have poor systemic resistance, usually combining with bacteremia or sepsis, systemic antibacterial therapy will be necessary for them. Therefore, it is necessary to conduct a comprehensive assessment of individual patients with diabetic foot ulcers, monitor the degree of infection and changes in pathogenic bacteria, and carry out anti-infective therapy in a standardized manner. At present, it is suggested that the treatment time of antibiotics for patients with mild foot infection should be 1 to 2 weeks, that for moderate and severe infection should be 2 to 3 weeks, and that for some can be extended to 4 weeks. It is not recommended to simply consider missing clinical symptoms as a sign of drug withdrawal because patients with diabetic foot ulcer lack of specificity of clinical symptoms. It should be comprehensively considered in combination with other clinical indicators [2].

In conclusion, this study found that patients diabetic foot ulcers are at high risk of infection with multi-drug resistant bacteria, and the assessment of the patient's condition after admission needs to take into account of their BMI, HbA1c, FPG, TG, ulcer course, ulcer size, peripheral neuropathy, peripheral vascular lesions, osteomyelitis, white blood cell count, neutrophil percentage, and use of antimicrobial drugs within the last 3 months. Peripheral blood leukocyte count and neutrophil percentage are not only indicators to evaluate the degree of infection, but also valuable in the early assessment of the risk of infection with drug-resistant bacteria. Further studies are needed to elucidate the mechanism of the effect of low immune cell levels on ulcer infection with multi-drug resistant bacteria.

## Data Availability

The datasets used and/or analysed during the current study available from the corresponding author on reasonable request.

## Abbreviations

MDR: Multidrug-resistant
DFI: Diabetic foot infection
BMI: Body mass index
FPG: Fasting plasma glucose
HbA1c: Glycated hemoglobin
TG: Triglycerides
TC: Total cholesterol
LDL-c: Low-density cholesterol
HDL-c: High-density cholesterol
